# Effects of supervised structured aerobic exercise training program on high and low density lipoprotein in patients with type II diabetes mellitus

**DOI:** 10.12669/pjms.331.11758

**Published:** 2017

**Authors:** Syed Shakil-ur-Rehman, Hossein Karimi, Syed Amir Gillani

**Affiliations:** 1 Syed Shakil-ur-Rehman, Principal/Associate Professor, Riphah College of Rehabilitation Sciences, Riphah International University, Islamabad, Pakistan. PhD Physical Therapy Student, University Institute of Physical Therapy, Faculty of Allied Health Sciences, University of Lahore, Lahore, Pakistan; 2 Hossein Karimi, Professor, University Institute of Physical Therapy, Faculty of Allied Health Sciences, University of Lahore, Lahore, Pakistan; 3 Syed Amir Gillani, Professor and Dean, Faculty of Allied Health Sciences, University of Lahore, Lahore, Pakistan

**Keywords:** Supervised Structured Aerobic Exercises Training (SSAET), Diabetes mellitus, Dyslipidemia, Hyperlipidemia

## Abstract

**Background and Objective:**

Hyperlipidemia and dyslipidemia are very common conditions among patients with Type-2 diabetes mellitus (T2DM) and associated with increased risk of coronary heart diseases. Physical activity and exercises along with medical management and dietary plan are common strategies to use for the management of deranged lipid profile in patients with T2DM. We aimed to determine the effects of supervised structured aerobic exercise training (SSAET) program on high and low density lipoprotein in patients with T2DM.

**Methods:**

This randomized control trial study was conducted at Riphah Rehabilitation Research Centre (RRRC), Pakistan Railway General Hospital (PRGH) Rawalpindi from 1^st^ January 2015 to 30^th^ March 2016. The inclusion criteria was Type-2 diabetes patients of both gender aged between 40 to 70 years. Patients with severe complications like coronary artery diseases (CAD), and other serious complications like diabetic foot, and severe knee and hip osteoarthritis (OA) were excluded from the study. A total of 195 patients diagnosed with T2DM were screened out and 102 were selected for the study as per the inclusion criteria. All participants were randomly assigned into two groups, experimental ‘A’ (n=51) and control ‘B’ (n=51). Patients in group A were treated with SSAET program of 25 weeks at 3 days a week in addition to routine medical management, while patients in Group-B were on their routine medications and dietary plan. Serum LDL, and HDL were tested at baseline and after 25 weeks. The data was analysed through SPSS 20.

**Results:**

Mean and standard deviation of LDL in group A (n=51) was 118.56±19.17 (pre) and 102.64±13.33 (post), while the mean and standard deviation for Group-B (n=51) was 116.50±18.45 (Pre) and 109.88±17.13 (post). Both groups showed improvement but, Group-A treated with SSAET along with RMM showed significantly higher (P Value ≤ 0.05) improvement as compared with group B treated with RMM alone. Mean and standard deviation of HDL in Group-A was 42.70±8.06 (pre) and 47.47±7.16 (post), while the mean and standard deviation of group B is 43.37±8.15 (Pre) and 44.41±7.91 (post). Both groups showed improvement but Group-A treated with SSAET program along with RMM showed significantly higher (P Value ≤ 0.05) improvement than group B treated with RMM alone.

**Conclusion:**

SSAET program along with RMM is more effective strategy for the management of deranged lipid profile in patients with T2DM.

## INTRODUCTION

Diabetes mellitus is associated with increased risk of atherosclerotic changes and may lead to coronary heart disease (CHD).^1^ Plasma lipid profile including, hypertriglyceridemia, low-high density lipoprotein (HDL) and cholesterol levels are frequently abnormal in Type-2 diabetes and that can cause macrovascular and microvascular complications.^2^ Type 2 diabetes accounts for 90- 95% of those with diabetes, previously referred adult onset diabetes or non insulin dependent diabetes.^3^ Dyslipidemia is associated with Type-2 diabetes mellitus and the most common patterns of dyslipidemia in diabetic patients are decreased HDL and elevated triglycerides.^4^

Because of the increasing prevalence, type 2 diabetes has become widespread epidemic. In 2007 almost 24 million American had diabetes, six million cases were undiagnosed. Currently, it is estimated that almost 60 million American also have prediabetes, a condition in which blood glues level is higher than normal.^5^

Statin is the drug of choice for hypercholesterolemia in patients with Type-2 diabetes mellitus but many of these patients fail to reach the target LDL levels.^6^ Exercise plays an important role in the prevention and control of Type-2 diabetes, insulin resistance, prediabetes, gestational diabetes mellitus and diabetes related health complications. Both aerobic and resistance training improve insulin action and also assist in the management of blood glucose levels, lipids, and blood pressure. Most people with type 2 diabetes can perform exercise safely as long as certain precautions are taken.^7^

There is strong experimental evidence that exercise especially aerobic training done in clinical practice is more effective as compared to resistance exercises in the management of Type-2 diabetes mellitus.^8^ There is strong experimental evidence that exercise can decrease the risk of developing Type-2 diabetes, through weight loss and metabolic regulation. However pharmacological treatment is often preferred as lifestyle interventions are difficult to maintain long term.^9^ The current study was designed to find the effect of supervised aerobic exercise on high and low density lipoprotein in patients with diabetes mellitus.

## METHODS

This randomized controlled trial was carried out at Riphah Rehabilitation Research Centre (RRRC), Pakistan Railway General Hospital (PRGH) Rawalpindi from 1^st^ January 2015 to 30^th^ March 2016. A cohort of 195 patients was screened out as per inclusion criteria of patients with Type-2 diabetes mellitus, both genders, and age ranged between 40 to 70 years, while patients with severe complications like coronary artery diseases (CAD), and other serious complications like diabetic foot, and severe knee and hip osteoarthritis (OA) were excluded from the study.

A pilot study was conducted on 20 patients and sample size was calculated by “Epitools” an online sample size calculator. The statistical parameters used for sample size calculation were mean of Insulin resistance (IR) in experimental group (0.4340), mean of IR in control group (0.6402), variance (0.137), confidence level (0.95) and power (0.8). The total Sample size calculated was 102 and 51 per group.

Finally 102 patients were selected and randomly place into experimental ‘A’ (n=51) and control ‘B’ (n=51) groups by lottery method.

To ensure anonymity and confidentiality a written consent was taken from all study participants in English and Urdu languages before enrolled in the study. Approvals were also taken from the ethical review committees of University of Lahore, and Riphah International University, Islamabad, Pakistan.

Patients in Group-A were treated with SSAET program of 25 weeks at 3 days a week in addition to routine medical management, while patients in Group-B were on their routine medications and dietary plan. The intervention in experimental group were applied by medically graded treadmill in 5 phases started from 10 minutes duration and 30 minutes per week with zero inclination (phase-1) and progressed to 50 minutes per session and 150 minutes per week with inclination 12 degree (phase-5) at normal speed determined by 20 meter distance test, detailed description is given in Table-I.

**Table-I:** Details of intervention in experimental group.

Phases (5 weeks each)	Single Session duration	Per week duration	Inclination	Speed
Phases 1	10 minutes	30 minutes	Zero	Normal
Phases 2	20 minutes	60 minutes	3	Normal
Phases 3	30 minutes	90 minutes	6	Normal
Phases 4	40 minutes	120 minutes	9	Normal
Phases 5	50 minutes	150 minutes	12	Normal

Serum LDL, and HDL were tested at baseline and after 25 weeks. Direct enzymatic colorimeteric method was used for the estimation of HDL and LDL. The data was analysed through SPSS version 20, and independent t test was applied on the basis of normality test. The means and standard deviation were calculated and further analysis was done to detect if there have been significant changes were present p value was set at 95% level of significance.

## RESULTS

Mean age of the study sample (n=102) was 54.38±8.24 years, while the mean age of Group-A was 53.74±8.75 and the mean age of group B was 55.08±7.67 years. Among 102 study sample 34 (33.3%) were male and 68 (66.7%) were female patients. Details of baseline characteristics are given in Table-II.

**Table-II:** Baseline characteristics of the study participant.

Parameters	Mean ± Standard Deviation	p-value
Age	54.38 (±08.24) years	0.001
BMI	29.94 (±5.09) kg/m^2^	0.001
Fasting Blood Glucose Level	272.3 (±24.17) mg/dl	0.001
HbA1c	8.2 (±1.7%)	0.001

Mean and standard deviation of LDL in group A (n=51) was 118.56±19.17 (pre) and 102.64±13.33 (post), while the mean and standard deviation of group B (n=51) was 116.50±18.45 (Pre) and 109.88±17.13 (post). Both groups showed improvement but Group-A treated with SSAET along with RMM showed significantly higher (P Value ≤ 0.05) improvement as compared with Group-B treated with RMM alone, detailed description is given in Table-III.

**Table-III:** Independent T test showing LDL.

Groups (N=102)	Group A (n=51)	Group B (n=51)	P-value (Independent T test)
Pre LDL(Mean±SD)	118.56±19.17	116.50±18.45	0.579
Post LDL(Mean±SD)	102.64±13.33	109.88±17.13	0.019

Mean and standard deviation of HDL in group A was 42.70±8.06 (pre) and 47.47±7.16 (post), while the mean and standard deviation of group B was 43.37±8.15 (Pre) and 44.41±7.91 (post). Both groups showed improvement but group A treated with SSAET program along with RMM showed significantly higher (P Value < 0.05) improvement than Group-B treated with RMM alone, detailed description is given in Table-IV.

**Table-IV:** Independent T test showing HDL.

Groups (N=102)	Group A (n=51)	Group B (n=51)	P value (Independent T test)
Pre HDL(Mean±SD)	42.70±8.06	43.37±8.15	0.579
Post HDL(Mean±SD)	47.47±7.16	44.41±7.91	0.044

## DISCUSSION

The current study confirmed the positive effects of supervised structured aerobic exercises over the six months (25 weeks) in diabetic patients with deranged HDL and LDL. Group-A was treated with SSAET program at three days a week for 25 weeks in addition to routine medical management, while group B were treated with routine medication and dietary plan.

Sahay BK and his colleagues in 2002 conducted a similar study in the management of diabetes mellitus through life style modification and concluded that exercise and dietary control are the most important primary therapeutic modalities for the management of diabetes mellitus. Exercise is especially very effective in Type-2 diabetes mellitus as it increases insulin sensitivity and normalise dyslipidaemia.^10^ Our results also showed significant improvements in normalizing deranged HDL and LDL levels in patients with Type-2 diabetes mellitus, while treated with SSAET program.

A study was conducted in 2014 by Mann S and his colleagues and they concluded that regular physical activity increased HDL levels, while maintaining LDL and triglycerides levels. It was also concluded that very high intensity aerobic exercises are required for improving and decreasing LDL levels. It was observed that increase in calorific expenditure is associated with intensity and duration of aerobic exercises, and positively influence HDL and LDL levels. 11 Our study also demonstrated that the participants underwent a long duration i.e. 25 weeks of supervised aerobic exercise and there was reduction in LDL and increase in HDL levels.

Another study was conducted by Kelley GA and his colleagues in 2006, to test the effects of a 12 weeks training program of resistance, aerobic exercises and combined exercises at moderate intensity for 30 minutes, five days a week on HDL and LDL. They found significant improvements in blood parameters especially in total cholesterol, HDL, and LDL levels.^12^ Our findings in the current study also suggested that aerobic exercises performed for long duration for 25 weeks give good results and there were significantly higher improvements in HDL and LDL levels.

Lavie CJ in 2015 conducted a study that concluded that physical activity and exercises decrease the risk of cardio vascular diseases by increasing HDL, decrease adiposity, and decreasing blood glucose level with high statistical significance and modest functional impact of 3-5% in patients with T2 DM.^13^ According to our study results along with improvement in other parameters, there is a significant increase in serum HDL level, which decreases the risk of cardiovascular diseases.

### Limitations of the Study

Pre and post assessment of the outcome measures. Secondly it was a single center based study.

## CONCLUSION

From the results of the current study, it can be concluded that SSAET program with RMM is more effective strategy compared with routine medical management in the management of deranged lipid profile in patients with T2DM. Effective management of deranged lipid profile can reduce CAD risk in patients with T2DM.
